# Postoperative Evaluation of Health-Related Quality-of-Life (HRQoL) of Patients With Lumbar Degenerative Spondylolisthesis After Instrumented Posterolateral Fusion (PLF): A prospective Study With a 2-Year Follow-Up

**DOI:** 10.2174/1874325001711011423

**Published:** 2017-12-11

**Authors:** S. Kapetanakis, G. Gkasdaris, T. Thomaidis, G. Charitoudis, E. Nastoulis, P. Givissis

**Affiliations:** 1 Spine Department and Deformities, Interbalkan Medical Center, Thessaloniki, Greece; 2 Papanikolaou Hospital, Thessaloniki, Greece; 3 First Orthopaedic Department of Aristotle University of Thessaloniki, Papanikolaou Hospital, Exohi, Thessaloniki, Greece

**Keywords:** Posterolateral fusion, Instrumentation, Degenerative spondylolisthesis, Quality of life, VAS, SF36

## Abstract

**Background:**

Several studies have compared instrumented PLF with other surgical approaches in terms of clinical outcomes, however little is known about the postoperative HRQoL of patients, especially as regards to degenerative spondylolisthesis.

**Methods:**

A group of 62 patients, 30 women (48,4%) and 32 men (51,6%) with mean age 56,73 (SD +/- 9,58) years old, were selected to participate in a 2-year follow-up. Their pain was assessed *via* the visual analogue scale (VAS) for low back pain (VASBP) and leg pain (VASLP) separately. Their HRQoL was evaluated by the Short Form (36) Health Survey (SF-36). Both scales, VAS and SF36, were measured and re-assessed at 10 days, 1 month, 3 months, 6 months, 12 months and 2 years.

**Results:**

VASBP, VASLP and each parameter of SF36 presented statistically significant improvement (p<0.01). VASBP, VASLP and SF36 scores did not differ significantly between men and women (p≥0.05). The most notable amelioration of VASBP, VASLP was observed within the first 10 days and the maximum improvement within the first 3 months. From that point, a stabilization of the parameters was observed. The majority of SF36 parameters, and especially PF (physical functioning) and BP (bodily pain), presented statistically significant improvement within the follow up depicting a very similar improvement pattern to that of VAS.

**Conclusion:**

We conclude that instrumented PLF ameliorates impressively the HRQoL of patients with degenerative spondylolisthesis after 2 years of follow-up, with pain recession being the most crucial factor responsible for this improvement.

## 1 INTRODUCTION

Lumbar degenerative spondylolisthesis is a common cause of low back pain and sciatica. While the conservative treatment of degenerative spondylolisthesis has remained unchanged, the number of surgical techniques has increased over the last two decades [1].

Posterolateral instrumented fusion (PLF) referring to the combination of lumbar laminectomy for decompression and transpedicular instrumentation using pedicle screws and rods for stability, is one of the two main approaches to spinal fusion; the other is interbody fusion (IBF). Several studies have compared these two surgical approaches in order to evaluate the efficacy of each one of them in terms of pain relief and clinical outcomes [2]. However, little is known in the relevant literature about the postoperative quality of life of patients after PLF, especially as regards to degenerative spondylolisthesis. The quality of life was estimated using both the SF-36 and the VAS score, instead of using the Oswestry Disability Index (ODI), because we wanted to assess the HRQoL in its various aspects.

Our primary hypothesis was that the postoperative low back pain, the radiating leg pain and also, the various aspects of the quality of life of patients with degenerative spondylolisthesis and spinal stenosis would be significantly improved after posterolateral instrumented fusion surgery.

## 2 MATERIAL AND METHODS

### 2.1 Patients

From January of 2012 to January of 2015, 80 patients were subjected to PLF and 62 of them were selected to participate in a prospective clinical study in order to evaluate the effectiveness of this specific surgical technique as regards to pain levels and quality of life. The surgical procedure included neural decompression by laminectomy and foraminotomy, spinal stabilization by posterolateral fusion, pedicular screw and rod instrumentation and the fusion was performed by applying a mixture of an autogenous bone graft and an allograft Fig. (**1**). The patients were recommended for surgical procedure after failing non-operative treatment.

The diagnosis of spinal stenosis was established by MRI. Preoperative plain radiographs of the lumbosacral spine were obtained for all patients. Radiographs were evaluated before surgery for the type of spondylolisthesis and severity of slip for each patient Fig. (**2**). Spondylolisthesis grading was recorded according to Meyerding’s scale [3].

The total of patients was referred to the same orthopaedic spine surgeon and all the procedures were performed at the same hospital. Patients agreed to participate in the study and signed a fully informed written consent. The study was approved by the medical council of the hospital.

Inclusion criteria (at least one of the following):

- Intermittent neurogenic claudication
- Deteriorating chronic low back pain + radiating leg pain (radiculopathy)
- Spondylolisthesis level I or II, in plain profile x rays
- Spondylosis' findings in MRI images in compliance with the clinical findings
- Compliance with one of the types of Adult Spinal Deformity (ASD) classification system [4]
- Fail of conservative treatment (medication + physiotherapy)

### 2.2 Exclusion Criteria

- Radiating leg pain as a single symptom
- Bone fracture
- Disc hernia as a single MRI finding
- Recent lumbar spine trauma
- Spine tumor or infection
- Isthmic spondylolisthesis (spondylolysis findings in CT and MRI images)
- Severe slippage (advanced third- fourth degree listhesis)
- Reoperation.

## 3 METHODS

62 patients were selected to participate in a 2-year follow-up. Their pain was assessed *via* the visual analogue scale (VAS, a 0–10 numerical rating scale), for low back pain and leg pain separately [5]. Their health-related quality of life was evaluated by using the Short Form (36) Health Survey (SF-36). Patients were asked to complete the measurements right before surgery. Both scales, VAS and SF36, were measured and re-assessed at 10 days, 1 month, 3 months, 6 months, 12 months and 2 years. Clinical and radiographic assessments were performed at each follow-up evaluating additionally the amelioration of listhesis, evidence for fusion and potential complication.

### 3.1 SF-36 Scoring Scale

The SF-36 scoring scale has 36 items. The item 2 is self-reported health changes and does not participate in scoring. The remaining 35 entries constitute 8 dimensions, physiological function (physical functioning, PF), physical function (role-physical, RP), bodily pain (bodily pain, BP), general health (general health, GH), energy (vitality, VT), social function (social functioning, SF), emotional function (role-emotional, RE) and mental health (mental health, MH). The higher the total score of all these 8 dimensions, the better the quality of life. If respondents answered less than half of the number of entries then their questionnaires were considered invalid. SF-36 has shown good reliability, consolidation validity, discrimination validity and criterion-related validity [6].

### 3.2 Statistical Analysis

The statistical analysis of this study was performed with the statistical package SPSS, version 16.00 (SPSS Inc, Chicago, IL). The p-value <0.05 was determined as statistically significant difference level. We used Wilcoxon test to compare the group values pre- and postoperatively, and the Mann-Whitney U-test to compare values between men and women.

## 4 RESULTS

After all inclusion and exclusion criteria were considered, we finally analyzed the results of 62 patients who underwent instrumented PLF, in an attempt to evaluate their low back pain (VASBP) and leg pain (VASLP) after surgery, as well as, their postoperative quality of life by measuring the parameters of the SF36 scoring scale as described above. All patients underwent the procedure successfully. There were no intra-operative complications. All selected patients successfully reached the end of the follow up. Thus, the percent of the 2-year follow up is 100%. From the patients included, 30 (48,4%) were women and 32 (51,6%) men. The mean age was 56,73 (SD +/- 9,58) years old. Concerning the postoperative complications, there were two cases of postoperative superficial wound infection and one case of temporary postoperative radicular pain. Out of 62 patients, 60 had good bony fusion demonstrated on postoperative X-ray A-P view. Only 3 had pseudoarthrosis at 6 monthly follow-up X-ray. The VASBP, VASLP and SF36 scores did not differ significantly between men and women (p≥0.05).

**Fig. (5):**
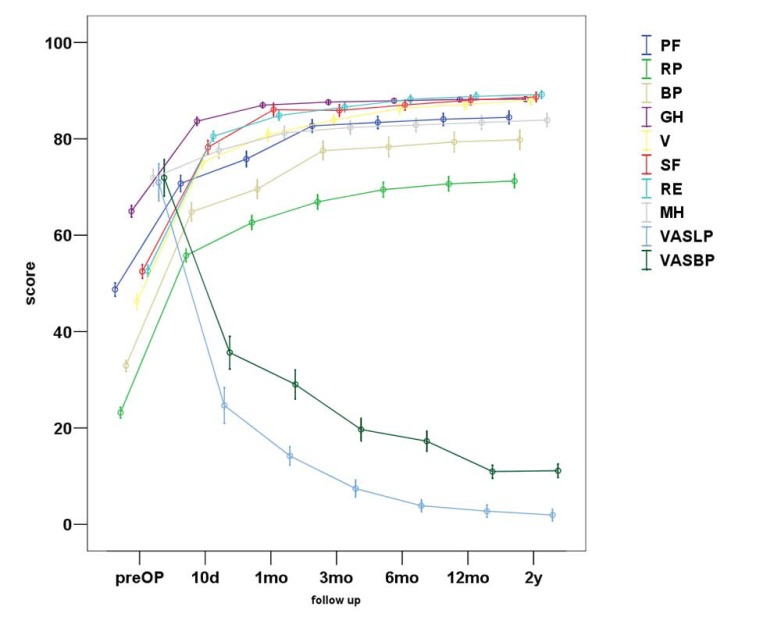
Graphic representation of the total VASLP and VASBP scores, as well as, the score of each parameter of the SF36 during the 2-year follow-up.

In total, VASBP, VASLP and each parameter of SF36 present statistically significant improvement (p<0.01). We can observe that the most notable amelioration of VASBP and VASLP respectively takes place within the first 10 days postoperatively, while the maximum result is observed almost within the first 3 months Figs. (**3**, **4**). During the second postoperative trimester we can see a slight improvement too. From that point to the end of our follow up limit (2 y), there is a stabilization of both our parameters. Physical functioning, role-physical, bodily pain, vitality, social functioning, role-emotional are the most improved parameters within the 2 years follow up, with the best results being observed within the first trimester and then being stabilized . As regards to the improvement of values, a similar pattern is observed between the VAS and the SF36 postoperative scores (Fig. **5**).

## 5 DISCUSSION

Decompression of the neural elements of the lumbar spine with laminectomy combined with instrumented PLF (transpedicular screws, rods, combined bone autologous graft and allograft) is a well-established and a time-tested surgical procedure for the treatment of lumbar spondylolisthesis. It has been stated that decompression mainly relieves radicular symptoms and neurogenic claudication, while fusion primarily relieves back pain by stabilizing the spine. Also, the addition of instrumentation leads to a solid arthrodesis [1]. Specifically, among patients with degenerative spondylolisthesis, the addition of lumbar spinal fusion to laminectomy has been associated with greater clinically improvement in overall physical health-related quality of life than laminectomy alone [7].

Based on our results, it was proven that lumbar decompression with laminectomy, combined with PLF provides very satisfactory clinical outcomes after 2 years of follow up. Great improvement was observed in VASBP and VASLP scores within 3 months postoperatively with leg pain almost disappearing in a 2-year time. From 3 months to 2 years the results remained almost stable (with a slow amelioration) and very satisfactory. The SF36 measured parameters related with the quality of life confirm the amelioration, especially the PF and BP improvement which reaches a very satisfactory result in 10 days time and stabilizes after the first year. Additionally, the other parameters of the SF36 show very satisfactory results within 10 days follow up as well. The results mentioned above indicate that pain and quality of life parameters are depended, so that the low back pain recession and the leg pain almost total extinction help patients to fulfill in some cases seamlessly not only their physical activities but different aspects of their life.

Various studies in the relevant literature have tested the efficacy PLF in comparison with PLIF as regards to lumbar spondylolisthesis. Luo *et al.* and Liu *et al.* comparative studies, showed moderate-quality evidence of PLIF procedures’ advantage in pain and satisfaction compared to PLF [8, 9]. However, both surgical fusion techniques (PLF and PLIF) appear to lessen the disability of patients with spondylolisthesis, and none of the fusion techniques has been related to a better outcome in terms of disability [10]. Taking under evaluation the satisfaction rate, physical function and radiological factors (bony fusion/pseudarthrosis), in a similar group of patients, decompression and PLF is proposed as the procedure of choice for degenerative lumbar listhesis, with the limitation of the short (6 months) follow up and the retrospective character of the study [1]. Concerning the type of spondylolisthesis, Omidi-Kashani *et al.* compared the radiological and clinical outcomes (VAS and ODI scores) of patients suffering from isthmic and degenerative spondylolisthesis and the results revealed that decompression and PLF improve significantly both pain and disability in both groups of patients [11]. Nevertheless, the literature is lacking in information regarding the postoperative quality of life after instrumented PLF.

Sometimes it is difficult to decide which is the appropriate surgical procedure in order to avoid certain pitfalls. PLIF has been proposed for high grade spondylolisthesis, which requires reduction or if the disc space is still high. When the slip grade is low, or the disc space is narrow, the PLF should be preferred [12]. For low-grade lumbar spondylolisthesis in mid-term follow-up, PLF shows loss of correction in most cases, but presents good clinical outcome and fusion rate [13]. Regarding a very common degenerative spondylolisthesis site which is L4-L5 level, it is mentioned that using both PLF and PLIF can ameliorate clinical symptoms when local stability is achieved [14]. Additionally, according to Nam *et al.*, when a multi-level fusion is considered, the proximal fusion levels should be carefully determined [15].

Newer techniques have emerged and the need for more comparative studies is obvious. It is considered that TLIF may be performed easily, safely and effectively with fewer complications for patients with lumbar spondylolisthesis [16]. Ghasemi compared TLIF versus instrumented PLF, and found that there were no significant differences in degenerative spondylolisthesis as regards to age, gender, Body Mass Index, smoking and comorbid conditions operation level, hospital stay and surgical complications. However, TLIF was shown to be superior to instrumented PLF with respect to functional outcome and fusion rate [17]. TLIF is considered to be superior to PLF in reduction of slippage and restoring disk height and might provide better improvement of leg pain. Nevertheless, the health-related outcomes are not significantly different between the two procedures [18]. Pooswamy *et al.* conclude that TLIF and instrumented PLF are equally efficacious options in the treatment of Grade I and II spondylolisthesis, except lytic type [19].

Ohtori *et al.* compared a group of patients suffering from degenerative spondylolisthesis who underwent PLF with those treated with ALIF and found that the last group had a better VASBP improvement after 2 years follow up (1,2 +_0,5 vs 2,7 +_ 0,6 respectively) [20]. PLF treated patients had the advantage of a shortened hospital stay and bed rest, while the bone fusion was similar. Our patients VASBP after 2 years was 1,1 +_ 0,55, similar to the ALIF group, while the advantage of the short bed rest and hospital stay remains. Newer and more technically difficult techniques including the lateral trans-psoas approach termed extreme, direct or lateral lumbar interbody fusion (XLIF, DLIF, LLIF) are gaining widespread popularity [21].

Minimally invasive spine surgery has recently gained a position in spinal fusion as well. Facet fusion has shown good clinical outcomes that might be superior to those of conventional PLF with a comparable fusion rate. Miyashita mentions that it is useful for managing degenerative lumbar spondylolisthesis and is a minimally invasive evolution of PLF [22]. The MIS-PLF utilizing a percutaneous pedicle screw system is less invasive compared to conventional open-PLF. The reduction in postoperative pain leads to an increase in activity of daily living, demonstrating rapid improvement of several functional parameters and thus, offering better mid-term results in terms of reducing low back pain and improving patients' daily living [23].

Several metanalyses have studied the efficacy of PLF in comparison with other procedure. Liu *et al.* compared circumferential fusion vs PLF in patients with spondylolisthesis and did not show significant difference between the two methods in clinical satisfaction. PLF can reduce complication rate and shortens operating time and CF has an advantage of restoring lumbar alignment and improving fusion rate in patients with isthmic spondylolisthesis [24]. It has been stated that patients with degenerative spondylolisthesis can be effectively managed with either a PLF or IBF with no significant differences in clinical outcomes or fusion rate between the two groups. Interestingly, PLF demonstrated a shortened hospitalization [25, 2]. Carreon *et al.* reported that substantial improvement can be expected in patients treated with fusion, regardless of technique, when an established indication such as spondylolisthesis or disc degeneration disease exists [26]. Recently, Challier *et al.* concluded that PLF is an efficient technique for the surgical treatment of degenerative spondylolisthesis. However, TLIF did not show its superiority neither in clinical nor alignment parameters despite a better fusion rate. As a result, it was suggested that TLIF is not mandatory in this specific indication [27].

The present study has several limitations. Due to the design, there was no proper control group because the objective of this study was not to emphasize on the comparison between PLF and other procedures, but to present the early results on the improvement of the quality of life of patients and the recession of pain. We did not want to include the widely used Oswestry Low Back Pain Questionnaire (ODI) or other questionnaires because we believe that SF-36 is a multi-scaled questionnaire which is more complete tool for quality of life including emotional and mental sections. In our opinion, SF36 in combination with VASBP and VASLP as research tools, are the most appropriate for this specific study and its purpose. Furthermore, it would increase the significance of our study results if we considered designing a follow-up lasting more than 2 years.

New studies focus on the results and advantages of MIS techniques, given that they reduce post-surgical pain, shorten hospitalization and minimize surgical time and complication rate. Despite the new trends in spine surgical therapies, open methods as PLF still remain the gold standard therapy in central spinal stenosis, due to the MIS methods limitations and their application on selective patients.

## CONCLUSION

Finally, we can conclude that, despite the advantages of PLIF procedures in spinal alignment and restoration, when we study patients suffering from degenerative lumbar spondylolisthesis, decompression of lumbar spine and PLF offers significant and remaining relief from the symptoms, while on the same time clearly upgrades the patients’ quality of life, so that it can be considered the surgical procedure of choice for this group of patients.

## Figures and Tables

**Fig. (1):**
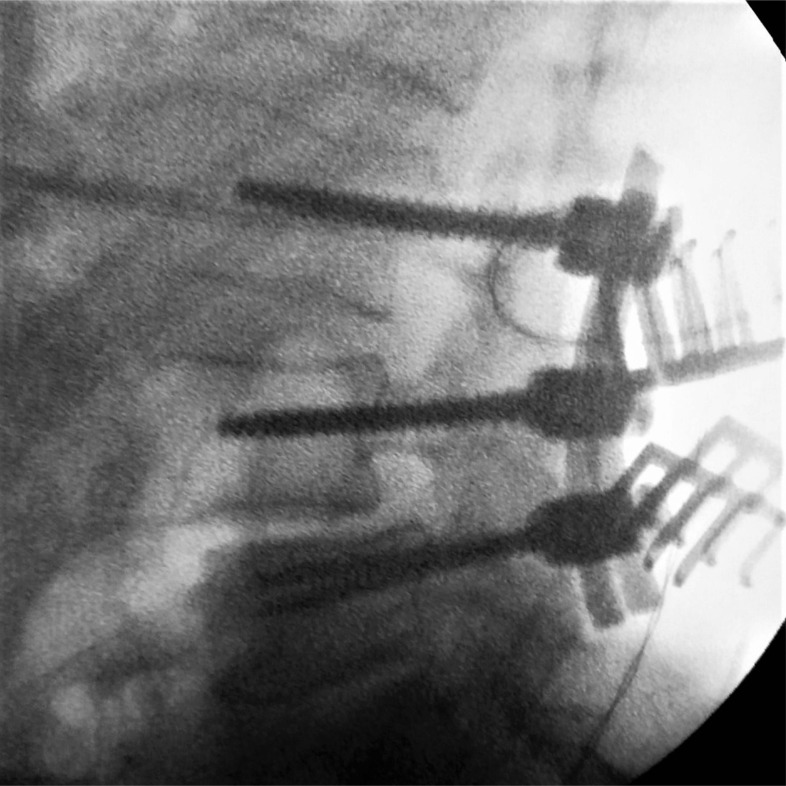
Intraoperative image of the C-arm depicting the instrumentation and spinal stabilization.

**Fig. (2):**
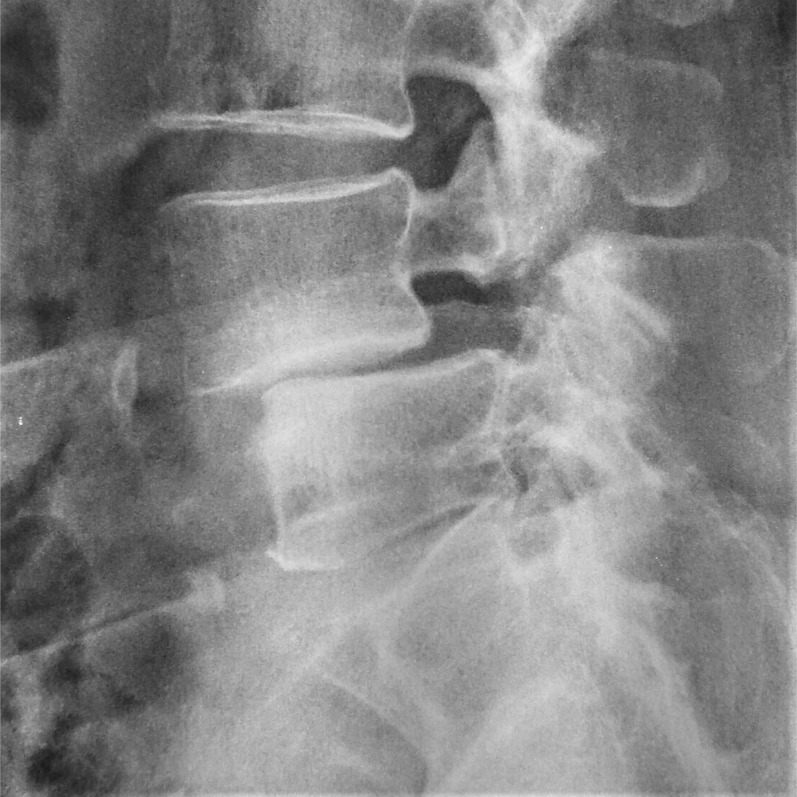
Profile X-ray of a patient with grade I-II spondylolisthesis of the L4 vertebra.

**Fig. (3):**
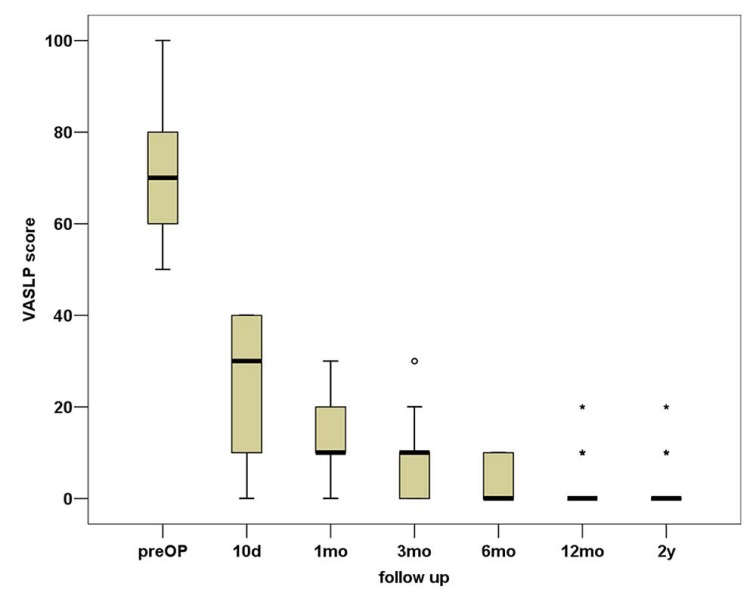
Box plot representation of the VASLP score during the 2-year follow-up.

**Fig. (4):**
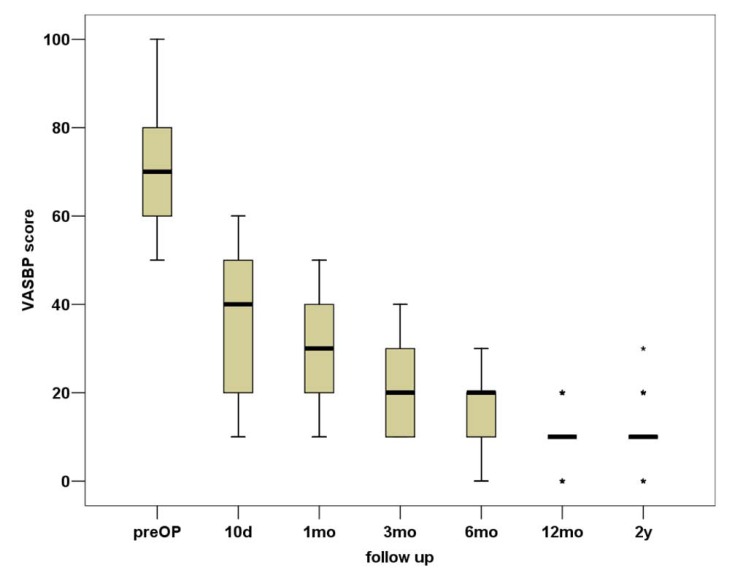
Box plot representation of the VASBP score during the 2-year follow-up.
